# Peptides extracted from edible mushroom: *Lentinus squarrosulus* induces apoptosis in human lung cancer cells

**DOI:** 10.1080/13880209.2017.1325913

**Published:** 2017-05-23

**Authors:** Arisara Prateep, Somruethai Sumkhemthong, Maneewan Suksomtip, Pithi Chanvorachote, Chatchai Chaotham

**Affiliations:** a Department of Biochemistry and Microbiology, Faculty of Pharmaceutical Sciences, Chulalongkorn University, Bangkok, Thailand;; b Department of Pharmacology and Physiology, Faculty of Pharmaceutical Sciences, Chulalongkorn University, Bangkok, Thailand;; c Cell-based Drug and Health Products Development Research Unit, Faculty of Pharmaceutical Sciences, Chulalongkorn University, Bangkok, Thailand

**Keywords:** Mushroom extract, selective anticancer activity, human safety, Bcl-2, BAX, c-FLIP

## Abstract

**Context:***Lentinus squarrosulus* Mont. (Polyporaceae) is an interesting source of diverse bioactive compounds.

**Objective:** This is the first study of the anticancer activity and underlying mechanism of peptides extracted from *Lentinus squarrosuls*.

**Materials and methods:** Peptides were isolated from the aqueous extract of *L. squarrosulus* by employing solid ammonium sulphate precipitation. They were further purified by ion-exchange chromatography on diethylaminoethanol (DEAE)-cellulose and gel filtration chromatography on Sephadex G25. Anticancer activity was investigated in human lung cancer H460, H292 and H23 cells cultured with 0–40 μg/mL of peptide extracts for 24 h. Cell viability and mode of cell death were evaluated by MTT and nuclear staining assay, respectively. Western blotting was used to investigate the alteration of apoptosis-regulating proteins in lung cancer cells treated with peptide extracts (0–20 μg/mL) for 24 h.

**Results:** The cytotoxicity of partially-purified peptide extracts from *L. squarrosulus* was indicated with IC_50_ of ∼26.84 ± 2.84, 2.80 ± 2.14 and 18.84 ± 0.30 μg/mL in lung cancer H460, H292 and H23 cells, respectively. The extracts at 20 μg/mL induced apoptosis through the reduction of anti-apoptotic Bcl-2 protein (∼0.5-fold reduction) and up-regulation of BAX (∼4.5-fold induction), a pro-apoptotic protein. Furthermore, *L. squarrosulus* peptide extracts (20 μg/mL) also decreased the cellular level of death receptor inhibitor c-FLIP (∼0.6-fold reduction).

**Conclusions and discussion:** This study provides the novel anticancer activity and mechanism of *L. squarrosulus* peptide extracts, which encourage further investigation and development of the extracts for anticancer use.

## Introduction

Lung cancer is currently a leading cause of cancer mortality, worldwide (Siegel et al. 2015). For several decades, attempts have been made to search for novel active compounds and strategies with improved efficacy and safety profiles (Bailly 2009). Although several available chemotherapies are currently prescribed for the treatment of lung cancer, their usage is frequently limited by severe side effects as well as drug resistance (Lemjabbar-Alaoui et al. 2015). Apoptosis induction is currently the most focused activity in anticancer-based natural product research. Apoptosis is a programed cell death that eliminates unwanted, harmful and damaged cells to maintain homeostasis and cell population (Samali et al. 2010). The deregulation or disruption of apoptosis leads to an aberrant of cell population, tumour initiation and eventually, cancer pathology (Brown & Attardi 2005). Therefore, apoptosis is considered a key mechanism for inhibition as well as elimination of cancer. For a mechanistic approach, apoptosis is regulated through two main pathways, which are intrinsic (mitochondrial) and extrinsic (death receptor). The intrinsic pathway causes the alteration in the balance of Bcl-2 family proteins such as Bcl-2 (B-cell lymphoma 2), Mcl-1 (Myeloid Cell Leukaemia 1), Bcl-xL (B-cell lymphoma-extra large) and BAX (BCL2 Associated X Protein) (Indran et al. 2011). The shift in cellular Bcl-2 family proteins toward an increase of pro-apoptotic proteins and/or a decrease of anti-apoptotic proteins results in the instability of mitochondrial membrane causing the release of cytochrome c to cytoplasm (Portt et al. 2011; Czabotar et al. 2014). The cytosol cytochrome c then stimulates caspase activation cascade (McDonnell et al. 2003). In terms of drug discovery, several natural product-derived compounds have been reported to exert anticancer activities by modulating the level of pro-and anti-apoptotic proteins of Bcl-2 family (Elmore 2007; Halim et al. 2011; Powan et al. 2013). An extrinsic apoptosis pathway induced by death ligand binding to its receptor, frequently fails to execute the cancer cells because of the high cellular level of c-FLIP (FLICE-like inhibitory protein), a potent inhibitor for caspase-8 (Wang et al. 2008; Safa & Pollok 2011). Taken together, targeting these anti-apoptosis members of the Bcl-2 family and c-FLIP are a promising way to sensitize the cancer cells to apoptosis.

As a unique natural source of diverse bioactive compounds, edible mushrooms are a potential resource for novel anticancer drug discovery. Apoptosis induction has been demonstrated in cancer cells cultured with peptides isolated from edible mushrooms (Patel & Goyal 2012; Dan et al. 2016). Due to its safety as a traditional food and its potential therapeutic effects, *Lentinus squarrosulus* Mont. (Polyporaceae) has been highlighted. Beside carbohydrates, proteins, vitamins and minerals, *Lentinus squrrosulus* also contains various bioactive compounds, including phenolic compound, immunostimulatory glucans and lectins peptide (Mhd Omar et al. 2011; Sen et al. 2013; Das et al. 2017). This study aimed to evaluate the anticancer activity and the underlying mechanism of peptide extracted from the Thai edible mushroom *Lentinus squarrosulus* in human lung cancer cells.

## Materials and methods

### Chemical reagents

Roswell Park Memorial Institute (RPMI) 1640 medium, phosphate-buffered saline (PBS) pH 7.4, trypsin, l-glutamine, foetal bovine serum (FBS) and penicillin/streptomycin solution were obtained from Gibco (Gaithersburg, MA). Prigrow III medium for human dermal papilla DPCs cells was purchased from Applied Biological Materials Inc. (Richmond, Canada). Hoechst33342, propidium iodide (PI), dimethysulphoxide (DMSO), ethyl alcohol, 3-(4,5-dimethylthiazol-2-yl)-2,5-diphenyltetrazolium bromide (MTT), TRIS hydrochloride (Tris–HCl), sodium chloride (NaCl), Tween 20, skim milk, bovine serum albumin (BSA) and ammonium sulfate were purchased from Sigma Chemical Inc. (St. Louis, MO). A bicinchoninic acid (BCA) protein assay kit was purchased from Thermo scientific (Waltham, MA). Protease inhibitor cocktail was obtained from Roche Molecular Biochemicals (Indianapolis, IN). Antibodies for Bcl-2, BAX, caspase-3, caspase-8, PARP, c-FLIP, β-actin and peroxidase-labelled secondary antibodies were obtained from Cell Signalling Technology Inc. (Denver, MA). Immobilon Western chemiluminescent HRP substrate was purchased from Millipore, Corp (Billerica, MA).

### Preparation of peptide extracts

#### Isolation of crude peptides

**Figure 1.**
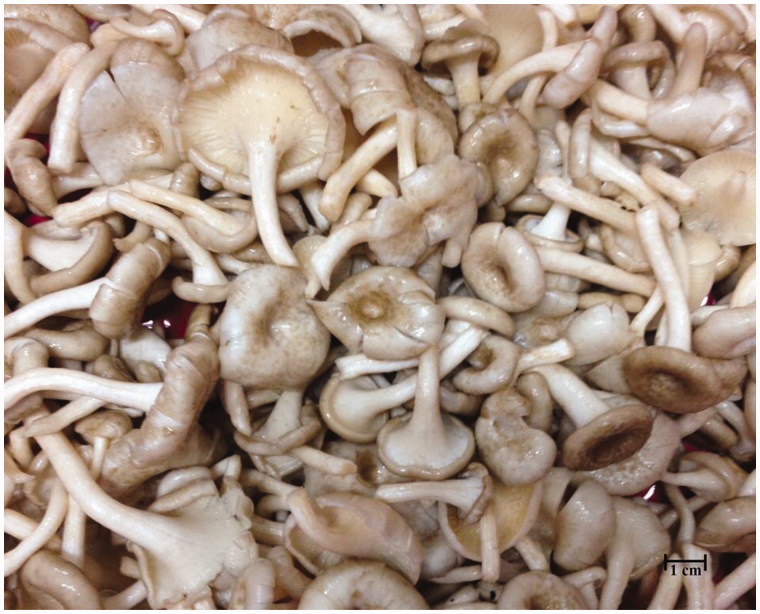
Thai edible mushroom, *Lentinus squarrosulus* Mont.

Partially-purified peptide extracts from *Lentinus squarrosulus* were prepared and kept in −80 °C. Briefly, fresh fruiting bodies of *L. squarrosulus* (Figure 1) were homogenized in deionized sterile water (3 mL/g). The clear supernatant was collected after centrifugation at 12,000 *g*, 4 °C for 30 min. Then, Tris–HCl buffer (pH 7.4) was added until the concentration reached 10 mM. Solid ammonium sulfate was slowly added to reach 40–80% saturation. After 1 h at 4 °C, the protein pellet was collected by centrifuge at 12,000 *g*, 4 °C for 30 min. The pellets were re-solubilized in 50 mL of 10 mM Tris–HCl buffer (pH 7.4) and dialyzed overnight with 10 mM Tris–HCl buffer at 4 °C to eliminate ammonium sulfate. Freeze-drying method was used to obtain the concentrated crude peptides.

#### Peptide purification

For further purification, ion exchange chromatography on diethylaminoethyl (DEAE)-cellulose (Sigma Chemical, St. Louis, MO) column (5 × 30 cm) pre-equilibrated with 10 mM Tris–HCl buffer (pH 7.4) was used. The bound peptides were eluted with step-wise salt concentration gradient (0, 0.1, 0.5 and 1 M NaCl) in 10 mM Tris-HCl buffer (pH 7.4) at a flow rate of 0.2 mL/min. The eluted fractions with highest absorbance at 280 nm were pooled and concentrated using freeze-drying. Further purification of these pooled fractions was carried out through size exclusion chromatography on Sephadex G25 (Amersham Bioscience, Piscataway, NJ) column (5 × 30 cm) pre-equilibrated with 10 mM Tris–HCl buffer (pH 7.4). The peptides were eluted with 10 mM Tris–HCl buffer (pH 7.4) of the previously described flow rate. All steps of purification were performed at 4 °C. Fractions with highest absorbance at 280 nm were pooled and concentrated using freeze-drying. The concentrated peptides were further determined for protein content, homogeneity, cytotoxicity, mode of cell death and western blot analysis.

#### Determination of protein content

The concentrated purified fractions of size exclusion chromatography were further determined for total protein content by a BCA assay kit to acquire an equal amount of peptides for further experiments. The concentrated purified fractions were freshly dissolved in deionized sterile water, then incubated with the mixer between BCA Reagent A and B at a ratio 50:1 in a dark place at 37 °C for 30 min. The optical density of the purple colour product was evaluated via microplate reader (Anthros, Durham, NC) at 562 nm. The protein concentration was calculated from the calibration curve of bovine serum albumin (BSA) at 0–12 μg/μL.

#### Evaluation on homogeneity of purified fractions

The homogeneity of purified fractions of size-exclusion chromatography was determined by sodium dodecyl sulfate-polyacrylamide gel electrophoresis (SDS-PAGE). The method was carried out as described by Laemmli and Favre (1973), using a 15% (w/v) gel (Laemmli & Favre 1973). The gels were stained with 0.1% Coomassie brilliant blue R-250 solution and destained with methanol: acetic acid: water (30:10:60% v/v) solution.

### Cell culture

All cell lines (passage number of 30–50) were cultured in an attachment cell culture plate at the optimum condition in the incubator supplied with 5% CO_2_ at 37 °C until they reached 70–80% confluence before using for further experiments. Human lung cancer H460, H292 and H23 cells (ATCC, Manassas, VA) were cultured in RPMI 1640 medium while human dermal papilla DPCs cells (Applied Biological Materials Inc., Richmond, Canada) were cultured in Prigrow III medium. The cultured mediums were replaced with fresh completed mediums supplemented with 2 mM l-glutamine, 10% FBS and 100 units/mL of penicillin/streptomycin every two days.

### Cytotoxicity assay

Cells were seeded onto a 96-well plate at a density of 1 × 10^4^ cells/well. After cultured for 12 h, the cells were further incubated with peptide extracts at 0–40 μg/mL for 0–24 h. At indicated time point, cell viability was determined by MTT assay. The cells were incubated with 0.4 mg/mL of MTT in a dark place at 37 °C for 4 h. Then, the supernatant was replaced with DMSO to dissolve the formazan product. The intensity of formazan colour was examined by microplate reader (Anthros, Durham, NC) at 570 nm. The optical density ratio of treated to non-treated control cells was calculated and presented in term of relative cell viability.

### Nuclear staining assay

Apoptotic and necrotic cell death were evaluated by co-staining of Hoechst33342 and propidium iodide (PI). After incubation with concentrated peptides (0–40 μg/mL) for 0–24 h, the cells at a density of 1 × 10^4^ cells/well in a 96-well plate were stained with 10 μM of Hoechst33342 and 5 μg/mL PI dyes for 30 min at 37 °C. The apoptotic and necrotic cells were visualized under a fluorescence microscope (Olympus IX51 with DP70) as condensed chromatin and/or fragmented nuclei and red fluorescence-positive cells, respectively.

### Western blot analysis

Cells were seeded at a density of 5 × 10^5^ cells/well onto a 6-well plate for 12 h and cultured in a completed medium containing peptide at 0–20 μg/mL for 24 h. After washing with cold PBS, the cells were incubated in lysis buffer containing 20 mM Tris-HCl (pH 7.5), 1% Triton X-100, 150 mM sodium chloride, 10% glycerol, 1 mM sodium orthovanadate, 50 mM sodium fluoride, 100 mM phenylmethylsulphonyl fluoride and a protease inhibitor cocktail for 45 min at 4 °C. Then, the supernatant was collected, and the protein content was determined using the BCA protein assay kit. An equal amount of 40 μg protein from each sample was denatured by heating at 95 °C for 5 min with Laemmli loading buffer and, subsequently, loaded onto a 10% SDS-PAGE. After separation, proteins were transferred onto 0.45 μM nitrocellulose membranes (Bio-Rad, Hercules, CA). The transferred membranes were blocked for 1 h in 5% nonfat dry milk in TBST (25 mM Tris-HCl pH 7.5, 125 mM NaCl and 0.05% Tween 20) and incubated with the appropriate primary antibodies, either Bcl-2 (dilution of 1:1000 in TBST containing 5% BSA), BAX (dilution of 1:1000 in TBST containing 5% BSA), caspase-3 (dilution of 1:1000 in TBST containing 5% skim milk), caspase-8 (dilution of 1:1000 in TBST containing 5% BSA), PARP (dilution of 1:1000 in TBST containing 5% skim milk), c-FLIP (dilution of 1:1000 in TBST containing 5% BSA) or β-actin (dilution of 1:1000 in TBST containing 5% BSA) at 4 °C overnight. Then, the membranes were washed three times with TBST for 5 min and incubated with horseradish peroxidase-labelled isotype-specific secondary antibodies (dilution of 1:2000 in TBST containing 5% skim milk) for 2 h at room temperature. The immune complexes were detected by enhancement with chemiluminescence substrate and quantified by analyst/PC densitometric software (Bio-Rad Laboratory, Hercules, CA).

### Statistical analysis

Mean data were averaged from three independent experiments. Statistical analysis was performed on SPSS Statistic 22 version (Armonk, NY) using one-way ANOVA followed by Bonferroni’s *post hoc* test. A *p*-value ≤0.05 was considered as statistically significant.

## Results

### Partially-purified peptide extracts isolated from Lentinus squarrosulus

**Figure 2.**
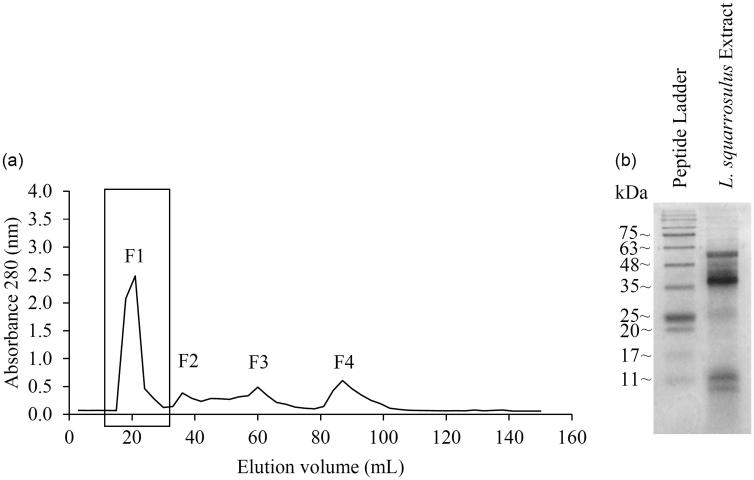
Isolated peptide extracts from *Lentinus squarrosulus*. (a) Peptide elution profile obtained from Sephadex G-25 gel filtration chromatography. The rectangular box indicates the highest peptide containing fraction (F1) which was used for further evaluation. (b) SDS-PAGE of F1 fraction of gel filtration chromatography.

Crude proteins were precipitated from a crude extract of *L. squarrosulus* fruiting-bodies with 40–80% (w/v) saturation of ammonium sulfate. The solubilized crude proteins were further purified by ion-exchange chromatography on a DEAE-cellulose column. After, the adsorbed protein was eluted with a step-wise salt concentration gradient of NaCl at 0, 0.1, 0.5 and 1 M in 10 mM Tris-HCl buffer (pH 7.4); only fractions eluted with 0.1 M NaCl showed the highest absorbance at 280 nm. They were then further purified by gel filtration on a Sephadex G-25 column. The elution profile of protein with a total four peaks, designated as F1 to F4, were obtained (Figure 2(a)). Sample from F1 with the highest absorbance at 280 nm was pooled and concentrated before further analysis. The SDS-PAGE analysis of the concentrated sample from F1 showed various bands in the size range of 10–50 kDa. Among these, there were four shape distinctions in size of around 9, 11, 38 and 56 kDa. The most intensive protein band was ≈ 38 kDa (Figure 2(b)). The recovery of protein in this step was about 0.159 ± 0.004% w/w.

### Cytotoxicity of the peptide extracts in human lung cancer cells

**Figure 3.**
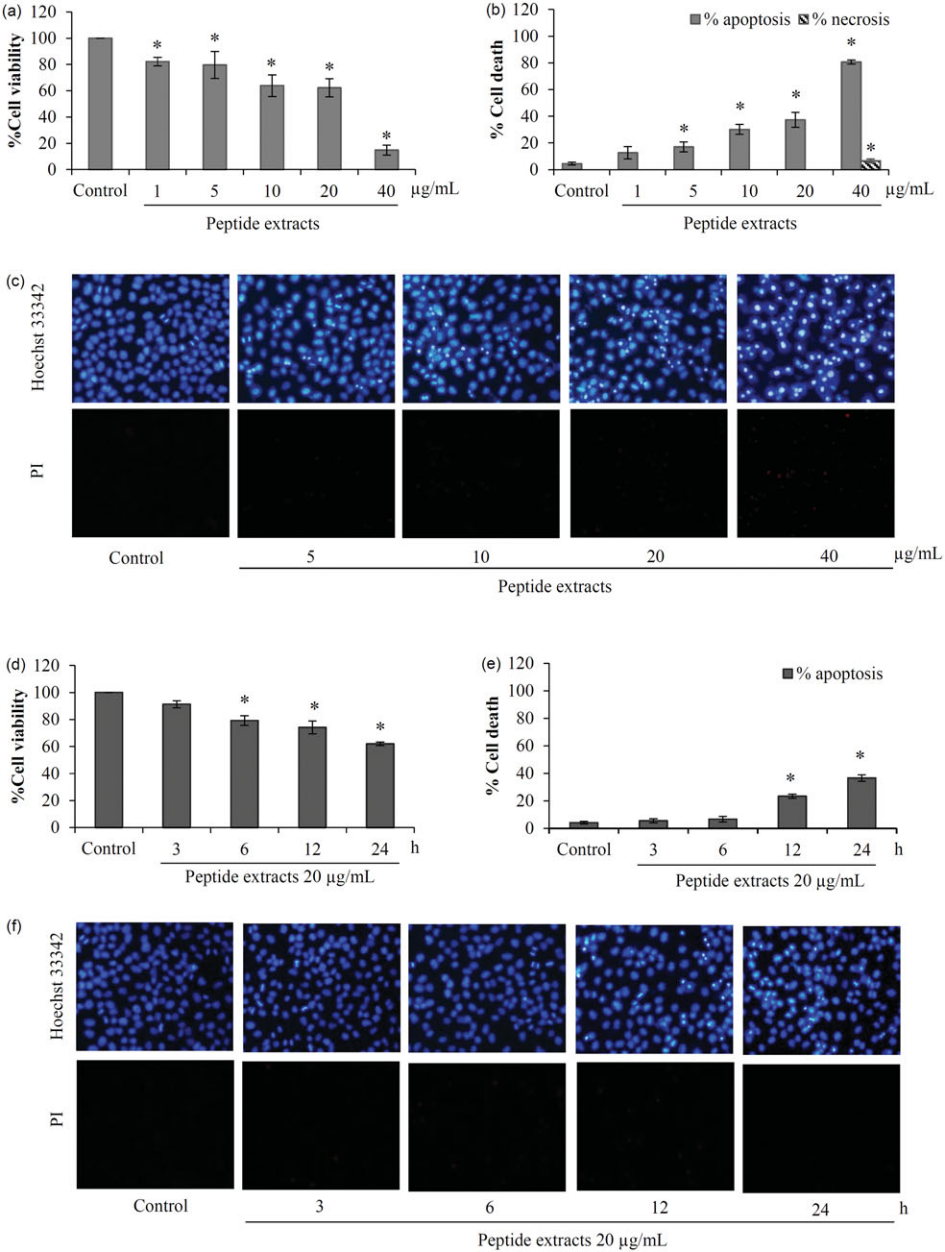
Apoptosis induction of peptide extracts from *Lentinus squarrosulus*. Cell viability of H460 lung cancer cells was evaluated after treatment with the extracts in (a) dose-dependent and (d) time-dependent manner. (b) There was a significant augmentation of apoptotic cell death in the incubation of peptide extracts at 5–40 μg/mL for 24 h. (e) Additionally, the effect of peptide extracts on apoptosis induction was early notified at 12 h of treatment of lung cancer cells with 20 μg/mL. (c and f) Apoptosis and necrosis cells were presented via co-staining of Hoechst33342 and propidium iodide. Values are means of the independent triplicate experiments ± SD. *p ≤ 0.05 versus non-treated control.

The reduction of cell viability was observed early at 6 h after treatment of H460 lung cancer cells with 20 μg/mL peptide extracts (Figure 3(d)). After 24 h of incubation, 40% reduction in viability of the cells was found in the cell treated with 20 μg/mL peptide extracts. Figure 3(a) presents dose-dependent cytotoxicity of the peptide extracts in human lung cancer cells. Notably, 85% of viable cell was suppressed after treatment of H460 cells with peptide extracts at the concentration of 40 μg/mL for 24 h.

Mode of cell death detected by co-staining of Hoechst33342/PI indicated apoptotic cell death after incubation H460 cells with the peptide extracts at 5–20 μg/mL for 24 h. Meanwhile, there was no indication of necrosis (Figure 3(b,e)). However, Figure 3(c) illustrated that the highest concentration (40 μg/mL) of peptide extracts caused both apoptosis and necrosis in human lung cancer cells with DNA condense stained by Hoechse33342 and red fluorescence of PI, respectively. Thus, peptide extracts at 5–20 μg/mL were selected for further evaluation of anticancer activity in human cancer cells.

### Apoptosis induction in lung cancer cells treated with peptide extracts

**Figure 4.**
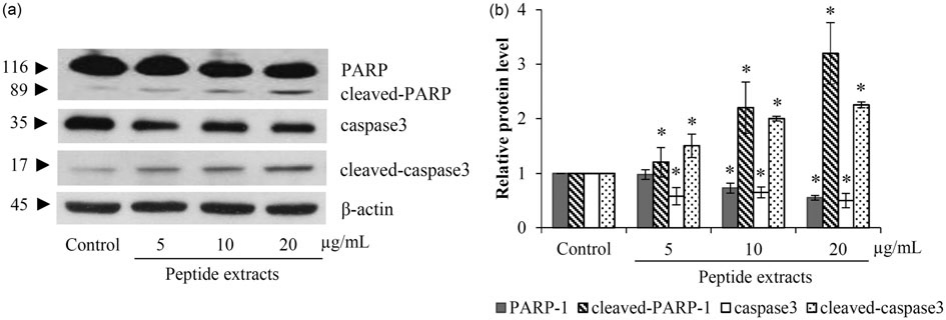
The apoptosis cells death was confirmed by the alteration of caspase-3 and PERP proteins. (a) Western blot analysis indicated an increase of active caspase-3 (cleaved- caspase-3) in peptide extracts-treated H460 cells. (b) As a substrate of activated caspase-3, the significant reduction of PARP was associated with the level of cleaved-caspase-3. Values are means of the independent triplicate experiments ± SD. *p ≤ 0.05 versus non-treated control.

To confirm peptide extracts-induced apoptosis, the evaluation on apoptotic marker proteins was performed via western blot analysis. The activation of caspase-3 indicating an increase of cleaved-caspase-3 was observed in treatment of H460 cells with peptide extracts (5–20 μg/mL) for 24 h (Figure 4). There was also significant reduction of PARP (Poly(ADP-ribose) polymerase-1) and the augmentation of cleaved-PARP in lung cancer cells treated with 10–20 μg/mL (Figure 4(b)).

### Peptides extracted from L. squarrosulus stimulate both intrinsic and extrinsic apoptosis pathways

**Figure 5.**
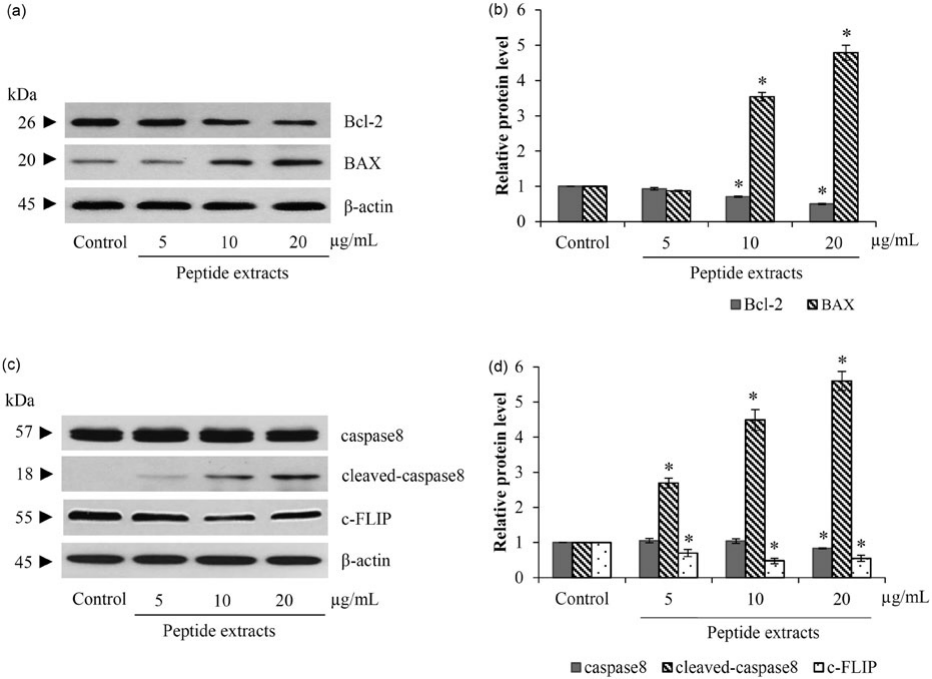
Peptide extracts stimulated both mitochondrial and death-receptor apoptotic pathway. (a and b) There were the reduction of Bcl-2 and up-regulation of BAX after treatment of lung cancer cells with 10–20 μg/mL of peptide extracts form *Lentinus squarrosulus*. (c and d) Protein-relating death-receptor pathway, c-FLIP and caspase-8 were decreased in peptide extracts-incubated H460 cells. Values are means of the independent triplicate experiments ± SD. *p ≤ 0.05 versus non-treated control.

There are two major apoptotic pathways, intrinsic and extrinsic machinery (Indran et al. 2011). The alteration of Bcl-2 family proteins involved in the intrinsic or mitochondrial pathway was significantly observed in H460 cells treated with the peptide extracts. Figure 5(a) shows that there was a reduction of Bcl-2, an anti-apoptotic protein, while a pro-apoptotic protein, BAX obviously increased after incubation of lung cancer cells with 10–20 μg/mL of peptide extracts for 24 h. The induction of extrinsic or death receptor signalling in peptide extracts-treated H460 cells was presented by the reduction of c-FLIP, an inhibitor of death receptor-activated caspase cascade which is associated with the augmentation of cleaved-caspase-8 (Figure 5(c)).

### Selective anticancer activity of peptides extracted from L. squarrosulus

**Figure 6.**
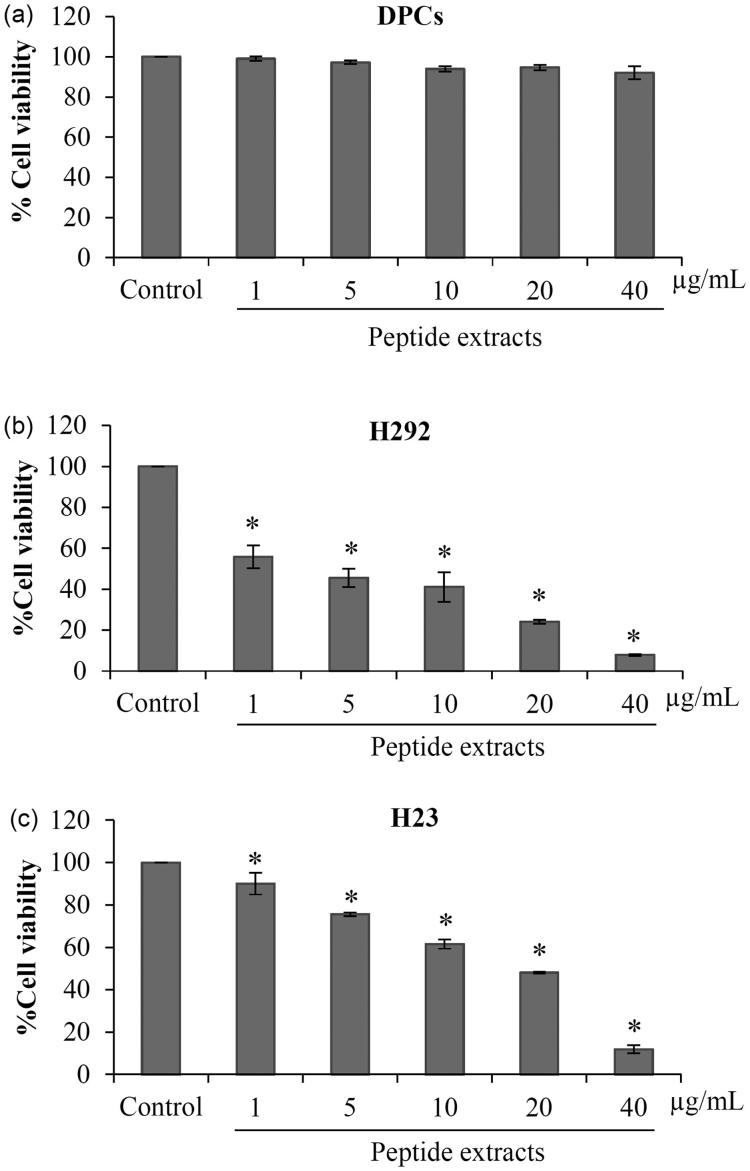
Selective anticancer activity of peptide extracts from *Lentinus squarrosulus*. (a) Low toxicity of the peptide extracts to non-cancer cells was indicated in human dermal papilla DPCs cells treated with 1–40 μg/mL peptides for 24 h. (b and c) Peptide extracts from *L. squarrosulus* induced cell death in various lung cancer cells. The anticancer activity of the extracts was also evaluated in H292 and H23 lung cancer cells. Values are means of the independent triplicate experiments ± SD. *p ≤ 0.05 versus non-treated control.

To evaluate the selective anticancer activity, cytotoxicity of the peptide extracts in human dermal papilla DPCs cells was investigated. Dermal papilla cells are one of the most affected normal cells induced by current chemotherapeutic drugs. Damage of dermal papilla cells leads to hair loss and a low quality of life in lung cancer patients (Herbst et al. 2004; Chie et al. 2004; Koizumi et al. 2016). The selective anticancer effect and human safety profile of *L. squarrosulus* peptides are presented in Figure 6. After 24 h of treatment, the peptide extracts at 1–40 μg/mL caused no significant alteration of % cell viability in DPCs cells compared with non-treated control cells (Figure 6(a)).

Due to various phenotypes of lung cancer cells (Boiarskikh et al. 2011), the anticancer effect of the peptide extracts was further examined in different types of human lung cancer (H292 and H23 cells). After 24 h of incubation with 1–40 μg/mL of peptide extracts, the cell viability of H292 and H23 obviously decreased (Figure 6(b,c)). Notably, H292 cells seem to be more sensitive to the peptide extracts than H460 and H23 cells. The half inhibitory concentration (IC50) was approximately of 26.84 ± 2.84, 2.80 ± 2.14, 18.84 ± 0.30 μg/mL for H460, H292 and H23 cells, respectively. These results confirm the effect of peptide extracts from *L. squarrosulus* against human lung cancer cells.

## Discussion

Among leading cancers, lung cancer is recognized as an important cause of cancer-related deaths, with a significant cause of death being failure of drug treatment (Dziedzic et al. 2016). Cancer research has focused on a search for novel active compounds with high efficacy and low toxicity. Thus, this study has demonstrated the promising anticancer effect of peptides extracted from the *L. squarrosulus* mushroom against human lung cancer cells. Although drug resistance in lung cancer is complex and frequently caused by several factors, the increase of anti-apoptotic proteins such as Bcl-2 and c-FLIP has been shown to be dominant (Indran et al. 2011). The over expression of Bcl-2 and accumulation of cellular Bcl-2 proteins have been shown to mediate lung cancer cell resistance to several chemotherapies as well as death stimuli (Wesarg et al. 2007; Yip & Reed 2008; Yang et al. 2009). Likewise, the increase in the level of c-FLIP has been shown to inhibit the death of cancer cells in response to immune cell-mediated apoptosis (Wang et al. 2008; Bagnoli et al. 2010; Safa & Pollok 2011). This investigation demonstrated that peptides extracted from *L. squarrosulus* at concentrations of 1–40 μg/mL significantly decreased viability and induced apoptosis of various human lung cancer cells including H460, H23 and H292 cells. The mechanism of apoptosis demonstrated in H460 cells showed that the peptide extracts mediated apoptosis by increasing BAX while decreasing Bcl-2 protein. The result from this study is consistent with others in which several natural-derived compounds have anticancer effects by suppressing Bcl-2 protein (Halim et al. 2011; Woo et al. 2012; Vizetto-Duarte et al. 2016). Although c-FLIP has been perceived to have a principle role as a death receptor-mediating apoptosis, studies also suggest that this protein increases cell survival and proliferation (Bagnoli et al. 2010; Dickens et al. 2012). For example, the increase of cellular c-FLIP has been shown to activate NF-κB (Baratchian et al. 2016), and inhibition of such a pathway by dominant expression of its inhibitory subunit IκB decreased cell survival (Chanvorachote et al. 2005). This study has shown that treatment of the cells with peptides extracted from *L. squarrosulus* results in a significant depletion of c-FLIP. Together with the above context, the extract may not only induce apoptosis, but also suppress cancer cell survival and proliferation. However, the efficacy of the peptides should be investigated in lung cancer cells with aggressive behaviours of chemotherapeutic resistance and metastasis characters.

Although many chemotherapeutic drugs are currently prescribed for the treatment of lung cancer, their usage is frequently limited by severe side effects to non-cancer cells (Lemjabbar-Alaoui et al. 2015). The high safety profile in humans of *L. squarrosulus* mushroom has been proven by the long time it has been consumed as a traditional food as well as a toxicity study of an animal model (Mhd Omar et al. 2011; Das et al. 2017). Selective anticancer activity presented in this study strengthens the potential development of extracts from *L. squarrosulus* with less side-effect than current chemotherapeutic drugs.

**Figure 7.**
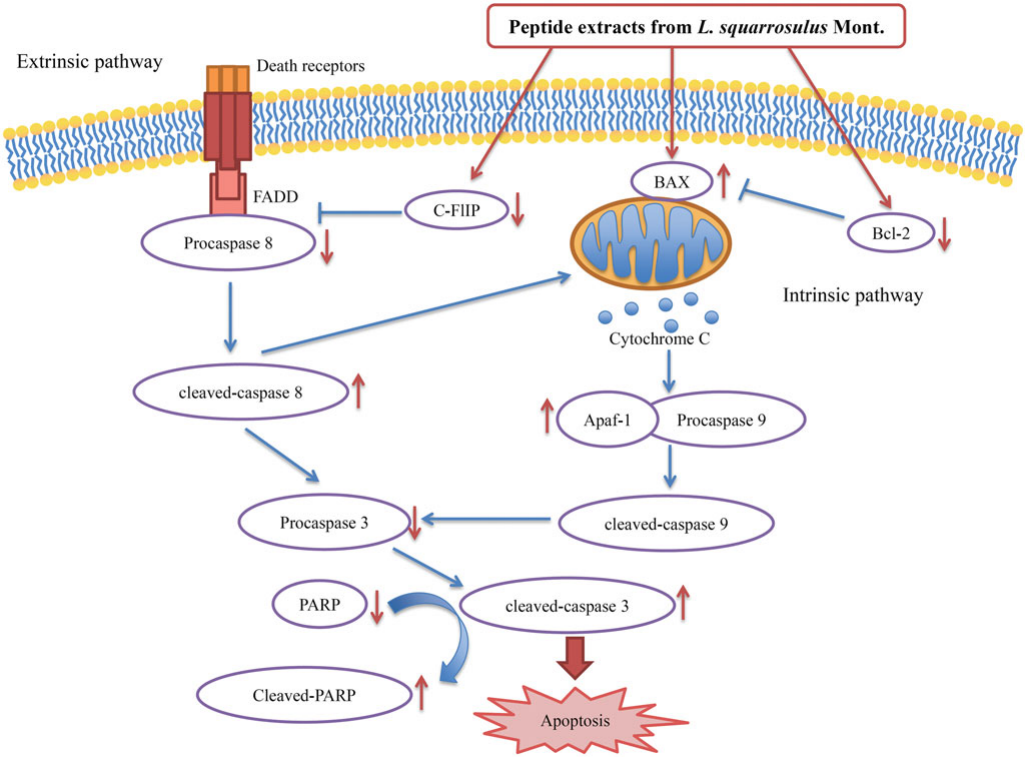
Proposed mechanistic scheme of anticancer activity of peptide extracts from *Lentinus squarrosulus* in human lung cancer cells. The extracts mediated a mitochondrial or intrinsic apoptotic pathway through the reduction of Bcl-2 and increase of BAX. Meanwhile, the extracts also decrease c-FLIP, an inhibitor for death-receptor regulating apoptosis.

In summary, the findings from this study highlight the potent effects of peptides extracted from fruiting bodies of *L. squarrosulus* mushroom, in mediating apoptosis in lung cancer cells through the decrease of anti-apoptotic Bcl-2 and c-FLIP proteins and increase of pro-apoptotic protein Bax (Figure 7). Nevertheless, the purification, examination and identification of amino acid sequencing of active peptide with a high efficacy and safety profile in extracts of *L. squarrosulus* should be further studied.
